# Inducing enhanced neutralizing antibodies against broad SARS-CoV-2 variants through glycan-shielding multiple non-neutralizing epitopes of RBD

**DOI:** 10.3389/fimmu.2023.1259386

**Published:** 2023-12-11

**Authors:** Qingyun Zhang, Yi Yang, Jun Lan, Ziyi Wang, Yan Gao, Xiao Li, Weidong Mao, Jing Xie, Li-Zhi Mi, Xiangyang Zhang, Xinquan Wang, Xin Mu, Kunrong Mei

**Affiliations:** ^1^ School of Pharmaceutical Science and Technology, Tianjin University, Tianjin, China; ^2^ School of Life Sciences, Tsinghua University, Beijing, China; ^3^ School of Biomedical Sciences, Hunan University, Changsha, China; ^4^ School of Life Sciences, Tianjin University, Tianjin, China; ^5^ Tianjin University and Health-Biotech United Group Joint Laboratory of Innovative Drug Development and Translational Medicine, Tianjin University, Tianjin, China

**Keywords:** SARS-CoV-2, RBD, glycosylation, glycan, subunit vaccine, neutralizing antibody

## Abstract

**Introduction:**

Since the outbreak of SARS-CoV-2, vaccines have demonstrated their effectiveness in resisting virus infection, reducing severity, and lowering the mortality rate in infected individuals. However, due to the rapid and ongoing mutations of SARS-CoV-2, the protective ability of many available vaccines has been challenged. Therefore, there is an urgent need for vaccines capable of eliciting potent broadly neutralizing antibodies against various SARS-CoV-2 variants.

**Methods:**

In this study, we developed a novel subunit vaccine candidate for SARS-CoV-2 by introducing a series of shielding glycans to the Fc-fused receptor-binding domain (RBD) of the prototypic spike protein. This approach aims to mask non-neutralizing epitopes and focus the immune response on crucial neutralizing epitopes.

**Results:**

All modified sites were confirmed to be highly glycosylated through mass spectrometry analysis. The binding affinity of the glycan-shielded RBD (gsRBD) to the human ACE2 receptor was comparable to that of the wildtype RBD (wtRBD). Immunizing mice with gsRBD when combined with either Freund’s adjuvant or aluminum adjuvant demonstrated that the introduction of the glycan shield did not compromise the antibody-inducing ability of RBD. Importantly, the gsRBD significantly enhanced the generation of neutralizing antibodies against SARS-CoV-2 pseudoviruses compared to the wtRBD. Notably, it exhibited remarkable protective activity against Beta (B.1.351), Delta (B.1.617.2), and Omicron (B.1.1.529), approximately 3-fold, 7- fold, and 17-fold higher than wtRBD, respectively.

**Discussion:**

Our data proved this multiple-epitope masking strategy as an effective approach for highly active vaccine production.

## 1 Introduction

The emergence of Severe Acute Respiratory Syndrome Coronavirus 2 (SARS-CoV-2) in late 2019 caused a global pandemic of Coronavirus Disease 2019 (COVID-19), resulting in severe respiratory illness and significant economic losses (1). The spread of SARS-CoV-2 continues to pose a significant threat to human health, with multiple variant strains identified and spreading worldwide (2–5). Given the ongoing threat, vaccines remain the most effective way to protect against SARS-CoV-2 infection and reduce the incidence of severe illness (6, 7).

SARS-CoV-2 encodes four structural proteins, including the spike (S), membrane (M), envelope (E), and nucleocapsid (N) proteins (8, 9). The S protein forms a trimeric complex responsible for binding to the host receptor angiotensin-converting enzyme 2 (ACE2) and facilitating membrane fusion during viral entry (10). Given its crucial role in receptor recognition and cellular invasion, the S protein, particularly its receptor- binding domain (RBD), represents the primary target for neutralizing antibodies and is the focus of vaccine design (11, 12). Notably, in a characterization of 377 human monoclonal antibodies, it was observed that nearly all highly inhibitory antibodies (IC50 < 0.1 μg/mL) bind to the receptor-binding motif (RBM) located at the top surface of RBD (13). These antibodies effectively block the interaction between RBD and ACE2, highlighting the RBM, particularly the ACE2 binding interface, as the pivotal neutralizing epitope. Moreover, all variants of SARS-CoV-2 still utilize ACE2 as the receptor for host cell entry (14, 15). Therefore, targeting the RBD, particularly the ACE2 binding interface, in antigen design remains an effective approach.

Glycosylation is an important post-translational modification process in which a glycan moiety is covalently attached to specific amino acid residues on proteins. Depending on the amino acids involved in glycan attachment, glycosylation is classified into N-glycosylation and O-glycosylation. N-glycosylation is prevalent in various organisms and occurs in the endoplasmic reticulum and Golgi apparatus. In this process, oligosaccharides are transferred by oligosaccharyltransferase to the side chain of asparagine (Asn) within the Asn-X-Ser/Thr sequence, where X represents any amino acid except proline (16, 17). There are three types of N-glycosylation modification based on the glycan linked to asparagine: high mannose structure, hybrid structure, and complex structure (18). On the other hand, O-glycosylation primarily occurs on the hydroxyl groups of serine, threonine, and tyrosine residues. Unlike N-glycosylation, O-glycosylation does not follow a conserved amino acid sequence, and the extent of glycosylation is more diverse and complex (19).

Glycosylation is a common strategy employed by enveloped viruses to evade immune detection. By forming a dense outer shell that covers the immunogenic protein surface, viruses such as HIV-1, influenza virus, and coronaviruses can effectively mask themselves from the immune system (20–22). This glycan masking approach has been adopted in the field of vaccinology to conceal regions of low importance and redirect the immune response towards highly therapeutic epitopes to ultimately achieve a more focused or broadened immune response (23). Research has shown that glycan masking of certain non-neutralizing epitopes on antigens of viruses such as HIV, the influenza virus, and the Middle East respiratory syndrome coronavirus (MERS-CoV) can effectively enhance neutralizing antibodies (24–27). The RBD of SARS-CoV-2 also contains numerous sites that do not contribute to the generation of neutralizing antibodies. Therefore, these sites are also worth considering for glycan masking.

In this study, we aimed to enhance the neutralizing ability of the receptor binding domain (RBD) of SARS-CoV-2 by introducing glycan modifications to specific epitopes outside the ACE2 binding interface. We successfully designed and expressed four RBD mutations in HEK293T cells, and confirmed them to be highly glycosylated. These mutations were then combined to create a modified RBD with a glycan shield, referred to as gsRBD. Immunization of mice with the gsRBD protein resulted in the production of RBD-specific antibodies comparable to those induced by wildtype RBD (wtRBD). However, serum from mice immunized with gsRBD exhibited significantly higher neutralizing activity against SARS-CoV-2 variant pseudoviruses compared to serum from wtRBD immunization. This finding suggests that the introduction of glycan shielding to non-neutralizing epitopes can enhance the generation of RBD-specific neutralizing antibodies against broad SARS-CoV-2 variants. Overall, our study provides a promising strategy to enhance the neutralizing antibody response against SARS-CoV-2 by glycan-shielding multiple non-neutralizing epitopes on the RBD. Furthermore, the gsRBD represents a subunit vaccine candidate with broad neutralizing potential against various epidemic SARS-CoV-2 variants.

## 2 Materials and methods

### 2.1 Cell lines

HEK293T cells were used for producing wild-type and mutant RBD-Fc proteins and SARS-CoV-2 pseudoviruses. HeLa cells expressing hACE2 were kindly provided by Dr. Qiang Ding from Tsinghua University and used as the target cells for the pseudovirus experiment. To outline the procedure, cDNA for hACE2 with a C-terminal FLAG-tag was synthesized and cloned into the pLVX-IRES-zsGreen1 vector (Catalog No. 632187, Clontech Laboratories, Inc). VSV-G-pseudotyped lentivirus expressing ACE2 tagged with FLAG was then produced and employed for transducing the HeLa cells (28). Both cell lines were cultured in Dulbecco’s modified Eagle’s medium (DMEM) supplemented with 10% fetal bovine sera (FBS) at a temperature of 37°C under 5% CO_2_. Sf9 cells, cultured in SIM medium at a temperature of 27°C and a rotation speed of 180 rpm, were used to express the extracellular domain of hACE2.

### 2.2 Animals

Specific pathogen-free (SPF) female BALB/c mice (6-8 weeks) were purchased from Beijing Vital River Laboratory Animal Technology Co., Ltd. (licensed by Charles River). All mice used in this study were in good health and had not been previously used in other experiments. The mice were housed at the Experimental Animal Service Platform of the Institute of Radiation Medicine, Chinese Academy of Medical Sciences and Peking Union Medical College. The mice had ad libitum access to standard food and water, and a 12-hour light-dark cycle was provided (temperature 20-25°C, humidity 50%-70%). Five mice were housed together per cage.

### 2.3 Construction, expression and purification of the RBD proteins

The DNA encoding RBD (residues 331-531) was inserted into a modified pcDNA3.1(+)-IL2-Sumo-Fc vector using the KpnI/BamHI restriction sites. To create the pcDNA3.1(+)-IL2-Sumo-Fc vector, synthesized DNA containing the Kozak sequence (GCCACC) and cDNA encoding the IL-2 signal peptide, 6× His tag, and SUMO tag was inserted into pcDNA3.1(+) via HindIII/KpnI restriction sites. Additionally, the human IgG1-Fc fragment, along with a stop codon, was further inserted using BamHI/XhoI sites. The inclusion of the Kozak sequence, IL-2 signal peptide and 6× His tag was aimed at enhancing the expression, secretion and affinity purification of the target protein. The SUMO tag can be recognized and cleaved by the Ulp1 protease, thereby facilitating the complete removal of the tag from the RBD-Fc protein. The BamHI restriction site between RBD and human IgG1-Fc encoded a GS linker to join them. Site-directed mutagenesis was performed on the wild-type RBD construct to introduce the desired glycosylation mutations. All recombinant plasmids were validated through Sanger sequencing.

For the expression of RBD proteins, HEK293T cells were used. The cells were seeded in 15 cm dishes one day prior to transfection. After 24 hours, the cell confluence reached approximately 90%. The recombinant plasmids (2 μg of DNA per mL of cell culture) were transfected into the cells using polyethylenimine (PEI) with a DNA : PEI ratio ranging from 1:2 to 1:3. After 6 hours, the medium was replaced with fresh medium, and the cells were further cultured for 66 hours before collecting the supernatant. Crude extract of the target protein was obtained by centrifugation to remove cell debris.

The target protein was initially purified using Ni-NTA beads. The supernatant from 200 mL of cell culture was filtered through 0.45-μm membranes and mixed with 10× Tris buffer (500 mM Tris, pH 8.0, 1.5 M NaCl) and 0.5 mL of Ni-NTA beads, followed by incubation at 4 °C for 2 hours. Non-specifically bound proteins were washed off the Ni-NTA beads using 1× Tris buffer (50 mM Tris, pH 8.0, 150 mM NaCl) supplemented with 20 mM imidazole. Subsequently, the target protein was eluted from the Ni-NTA beads using 1× Tris buffer containing 300 mM imidazole. The 6× His and SUMO tag were removed by adding Ulp1 (0.1 mg/mL) to the elution and incubating at 4°C for 1 hour. The protein was further purified using a Superdex 200 10/300 GL column, and the purity of the target protein was assessed using SDS-PAGE.

### 2.4 Expression and purification of hACE2

The hACE2 protein was expressed and purified in Sf9 cells using the Bac-to-Bac baculovirus expression system (Invitrogen). When the Sf9 cell density reached approximately 1.8×10^6^, the cells were seeded in a 6-well plate at a confluence of around 80%. Following cell adhesion, 4 μL of Bacmid (100-300 ng/μL) containing the gene encoding the extracellular domain of hACE2 was transfected into one well of Sf9 cells using X-tremeGENE™ 9 DNA Transfection Reagent. After 5 hours of incubation at 27°C, the medium was replaced with fresh medium. Following 7 days, the supernatant containing P0 baculovirus was collected. For protein expression, P2 baculovirus was obtained after two passages of P0 in Sf9 cells. P2 baculovirus was added to Sf9 cells at a volume ratio of 1:100, and the supernatant was collected after 72 hours. The supernatant was filtered, and the target protein was enriched using Ni-NTA beads. Subsequently, purification was performed using a Superdex 200 10/300 GL column. The purity of the target protein was assessed using SDS-PAGE.

### 2.5 Glycosylation analysis of RBD mutations by LC-MS

LC-MS was used to detect the glycosylation of RBD mutations. Initially, the N-glycans on the mutated RBD-Fc proteins were removed by treatment with 0.1 mg/mL PNGase F at room temperature for 16 hours. The deglycosylated RBD-Fc mutant proteins were separated by 12% SDS-PAGE. The gel bands containing deglycosylated glycoproteins were excised, subjected to destaining and drying, and subsequently treated with 25 mM DTT at 55°C for 45 minutes for reduction. After drying, 55 mM IAA was added, and the mixture was incubated at room temperature in the dark for 20 minutes for alkylation. The gel fragments containing a single glycosylation mutation of RBD-Fc were further treated with Trypsin working solution (Promega, 0.125 μg/mL), while the gsRBD was subjected to digestion with both Trypsin (0.125 μg/mL) and Chymotrypsin (0.125 μg/mL) working solutions. After incubation with Trypsin/Chymotrypsin at 4°C for 20 minutes, the centrifuge tube was inverted and further incubated at 37°C for 16 hours. The digested peptides were analyzed using a Fusion Lumos mass spectrometer coupled with an Easy-nLC 1200 system (Thermo Fisher Scientific). Peptides were loaded onto a 150 μm × 2 cm self-packed C18 trap column (particle size 3 μm, Dr. MASCH GmbH, Germany) and separated on a 150 μm × 30 cm self-packed C18 analytical column (particle size 1.9 μm, Dr. MASCH GmbH). The separated peptides were analyzed in MS. The MS operated in the data-dependent acquisition mode using Xcalibur 4.0 software, with a single full-scan mass spectrum in the Orbitrap (350–1500 m/z, 120,000 resolution), followed by data-dependent MS2 scans at 35% collision energy in the orbitrap with 30,000 resolution.

MS spectra from each LC-MS run were searched against the Swiss human protein database (released in October 2020) containing 20,311 sequence entries using the Proteome Discoverer (Version 2.2) searching algorithm. The search criteria included full tryptic specificity, allowance for two missed cleavages, fixed modification of carbamidomethylation (C), dynamic modification of oxidation (M), precursor ion mass tolerance of 20 ppm for all MS acquired in the Orbitrap mass analyzer, and fragment ion mass tolerance of 0.02 Da and glycosylation modified 0.984 Da (N) for all MS2 spectra acquired in the orbitrap (depending on whether it was glycosylated or not). A high confidence score filter (FDR < 1%) was used to select the “hit” peptides, and their corresponding MS spectra were manually inspected.

In addition to effectively removing almost all N-glycans, PNGase F treatment also deaminates the asparagine residue to aspartic acid, resulting in an increase in molecular weight compared to unmodified proteins. The abundance and intensity of the modified RBD-Fc mutants were compared to unmodified RBD-Fc. The relative abundance of glycosylated RBD-Fc mutants was represented using a bar chart in GraphPad Prism 8.0.

### 2.6 Interaction analysis between RBD and hACE2 using size exclusion chromatography

Purified RBD proteins and hACE2 were mixed at a molar ratio of 2:1, followed by incubation on ice for 2 hours. Subsequently, the sample was centrifuged at 16,200 g at 4°C for 10 minutes. The supernatant was collected and subjected to size exclusion chromatography (SEC) analysis using a Superdex 200 10/300 GL column. The eluted protein samples were examined using SDS-PAGE.

### 2.7 Interaction analysis between RBD and hACE2 using surface plasmon resonance

hACE2 was immobilized on a CM5 sensor chip using NaAc (pH 4.5) buffer, resulting in an immobilization level of approximately 216 response units. Subsequently, the RBD proteins were serially diluted with buffer containing 10 mM HEPES, pH 7.4, 150 mM NaCl from 800 nM to 50 nM and flowed over the CM5 chip. The experiment was carried out using a program consisting of a 90-second loading phase, followed by a 120-second dissociation phase. After each run, the chip surface was regenerated using 5 mM NaOH for 10 seconds. The flow rate during each run was set at 30 μL/min. The SPR curves obtained were fitted, and binding affinity values were calculated using Biacore Evaluation Software.

### 2.8 ACE2 expression and RBD binding detection via FACS

To detect ACE2 expression, the HeLa cells were treated with 0.5 mM EDTA, collected, washed twice with PBS, and then stained with 7-AAD at room temperature for 30 minutes. Subsequently, the signal of zsGreen1 and 7-AAD in the treated cells were analyzed using a flow cytometer (BD, FACSAria III/FACSVerse). Data analysis was performed using FlowJo 10.8.1.

The interaction between surface ACE2 and RBD was assessed following previously published methods (28). After ACE2-expressing HeLa cells were treated with 0.5 mM EDTA and collected, the cells were washed twice with cold PBS. Subsequently, the cells were incubated with RBD proteins (1 μg/mL) at 4°C for 30 minutes. After incubation, the cells were washed twice with cold PBS and stained with goat anti-human IgG (H+L) conjugated to Alexa Fluor 647 (Thermo Fisher, catalog no. A21445; 2 μg/mL) at 4°C for 30 minutes. Following this, the cells were washed twice with PBS and analyzed using a flow cytometer. Data analysis was performed using FlowJo 10.8.1 and GraphPad Prism 8.0.

### 2.9 Mice immunization

The gsRBD and wtRBD proteins were prepared in PBS buffer at a concentration of 0.25 mg/mL. To prepare the antigen-adjuvant mixture, each protein was thoroughly mixed with an equal volume of either Alum adjuvant or Freund’s adjuvant. The immunogenicity test consisted of six groups: gsRBD-Alum adjuvant group, wtRBD-Alum adjuvant group, gsRBD-Freund’s adjuvant group, wtRBD-Freund’s adjuvant group, and two PBS control groups (one for each adjuvant). Each group comprised five mice.

For the immunization process, each mouse received an injection of 80 μL of the antigen-adjuvant mixture. Specifically, 20 μL was injected into the abdomen, while 60 μL was injected into multiple points on the back and legs. Booster injections were administered 28 days after the primary immunization. Blood samples were collected from each mouse on day 21 after the first immunization, as well as day 7 and day 21 after the booster injection. Retro-orbital bleeding was employed for blood collection. The collected blood samples were incubated at room temperature for 2 hours and then centrifuged at 900 g for 15 minutes at 4°C. Subsequently, the supernatant was aliquoted and stored at -80°C for further analysis.

### 2.10 Detection of serum antibodies against RBD using ELISA assay

The ELISA plates were coated with 2 μg/mL of RBD in CBS buffer (carbonate-bicarbonate buffer) and incubated overnight at 4°C. After coating, the plates were washed three times with PBST (PBS with 0.05% Tween 20) to remove any unbound antigen. Blocking was performed by adding blocking buffer (1× PBS with 5% skim milk) to the plates and incubating at room temperature for at least 2 hours. Subsequently, the plates were washed three times with PBST.

For antibody detection, mouse sera were diluted 10,000-fold or serially diluted 4-fold in PBS buffer and added to the plates. The plates were then incubated at 37°C for 1 hour to allow antibody binding to the immobilized RBD. After incubation, the plates were washed three times with PBST. Next, HRP-conjugated goat anti-mouse IgG (Abclonal, 1:7500 v/v) was added to the plates and incubated at 37°C for 1 hour. Following incubation, the plates were washed three times with PBST. To visualize the antibody-antigen interaction, TMB (3,3’,5,5’-tetramethylbenzidine) working solution was added to the plates, and the enzyme reaction was allowed to proceed for 5-30 minutes at room temperature in the dark. The reaction was then stopped by adding 0.5 M H_2_SO_4_. The optical density at 450 nm, referred to as OD (450nm), was measured using a TECAN spectrophotometer. Each sample was measured in triplicate. The end-point titer values were determined based on the final dilution with an OD (450nm) value 2.1-fold higher than the negative control. Data analysis was performed using GraphPad Prism 8.0. The GMT (geometric mean titer) value of the titers was calculated with SPSS 27.0.1.

### 2.11 Pseudovirus neutralization assay

The pseudovirus was produced according to previously published methods (29–31). HeLa cells expressing hACE2 on their surface were utilized as the target cell, and cell entry efficiency of the pseudovirus was quantified by measuring luciferase activity.

To generate the pseudoviruses, the pNL4-3-R-E-luciferase vector (at a final concentration of 2.4 μg per mL of cell culture) and the pcDNA3.1 plasmid containing the full-length gene encoding either wildtype or variant SARS-CoV-2 S proteins (at a final concentration of 0.6 μg per mL of cell culture) were co-transfected into HEK293T cells using PEI when the cell confluence reached approximately 90% in 10-cm dishes. After 48 hours, the supernatant was collected by centrifugation at 3500 rpm for 10 minutes, yielding the SARS-CoV-2 pseudovirus. The pseudovirus was aliquoted and stored at -80°C until use.

For the neutralization assay, we followed previously established protocols (30, 31). Briefly, mouse sera were serially diluted 2.5-fold in DMEM supplemented with 10% FBS, starting at a dilution of 1:250. The diluted sera were added to 96-well plates. Subsequently, 100 μL of SARS-CoV-2 pseudovirus was added to each well and incubated at 37°C for 1 hour. Next, freshly trypsin-digested HeLa cells (20,000 cells per well) were added and incubated at 37°C and 5% CO_2_ for 48 hours. Following the incubation, the supernatant was removed, and 100 µL of Bio-Glo™ Luciferase Assay System was added to each well. After a lysis period of 5-10 minutes, luciferase activity was measured using a TECAN spectrophotometer. To determine the 50% pseudovirus neutralization titer (pVNT_50_), the sera dilution required to neutralize 50% of the pseudovirus was calculated by fitting a non-linear regression curve using GraphPad Prism 8.0. The GMT (geometric mean titer) value of pVNT_50_ was calculated with SPSS 27.0.1.

### 2.12 Statistical analysis

Statistical analysis was performed using Graphpad Prism 8.0 software to analyze the data obtained in this study. For the results of RBD proteins binding to ACE2 expressed in HeLa cells, the significance of differences between the groups was determined using one-way ANOVA followed by Dunnett’s multiple comparisons tests. Graphs illustrating the titers of antisera and pVNT_50_ of pseudovirus were generated. Regarding the titers data and pseudovirus results, two-tailed Mann-Whitney tests were conducted to determine whether there were statistically significant differences between the various groups. For the comparisons of multiple groups at different time points, the significance of differences between the groups was determined using one-way ANOVA followed by Tukey’s multiple comparisons tests. In this study, P ≤ 0.05 was considered to indicate statistical significance.

## 3 Results

### 3.1 Design of SARS-CoV-2 RBD antigen with glycan modification

**Figure 1:**
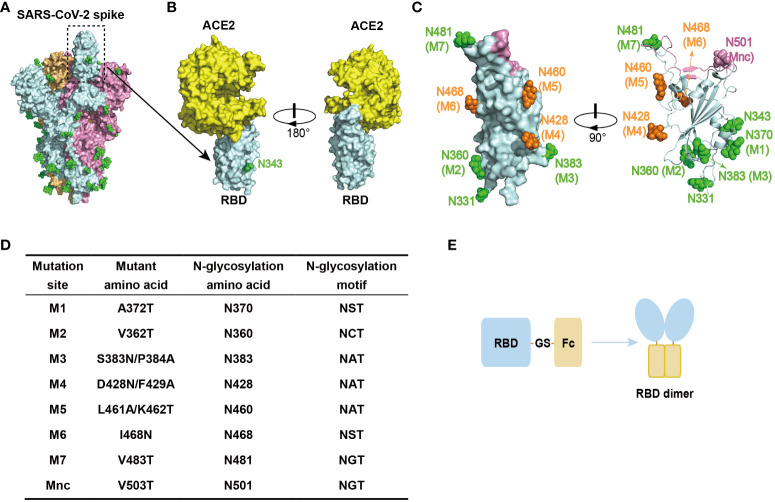
Design of RBD antigen with glycan modifications. **(A)** Schematic representation of the trimeric structure of the S protein of SARS-CoV-2 (PDB: 6VSB), highlighting the “up” conformation of the RBD. Each S protein monomer is depicted in a different color, and the green spheres on the surface indicate native N-glycosylation modifications. **(B)** Schematic representation of the interaction between RBD and hACE2 (PDB: 6M0J). The N343 glycan modification is indicated. **(C)** Schematic representation of the glycan modifications designed for RBD, including the two native glycan modifications at N331 and N343. The glycan modifications present in gsRBD (glycan-shield RBD, discussed later in the text) are depicted in green. Glycan mutations that failed to express are shown in orange. The negative control, Mnc, is marked in pink. The ACE2 binding interface is shown in pink. **(D)** The sites of glycan modifications and the corresponding mutations. **(E)** Schematic representation of the RBD antigen design. The human IgG1 Fc fragment is linked to the C-terminus of the RBD, facilitating RBD dimer formation through a disulfide bond between the Fc dimers.

RBD is located on the spike protein of SARS-CoV-2, which binds to the human ACE2 (hACE2) receptor and mediates virus entry into host cells (32). It is thus the primary target for neutralizing antibodies (**Figures 1A, B**) (11, 13), making it an ideal candidate antigen for a COVID-19 subunit vaccine in this study. To minimize the induction of non-neutralizing immune responses, we introduced N-glycosylation modification sites into the RBD to create glycan chains that would mask non-neutralizing epitopes. These glycan modifications were strategically placed on loops of the RBD surface, away from the ACE2 binding interface, to preserve the RBD structure and its interaction with ACE2. Additionally, the glycan modifications were evenly distributed to ensure that they did not interfere with each other and could effectively shield a larger non-neutralizing surface when combined.

Following this design principle, we engineered seven glycosylation mutations (M1-M7) on the SARS-CoV-2 RBD (residues 331-531) (**Figures 1C, D**). In addition to these artificial glycosylation sites, there are two native N-glycosylation sites at N331 and N343 of the RBD, which are naturally highly glycosylated sites (10, 33). Consequently, the introduction of these seven artificial glycosylation mutations theoretically results in the presence of nine glycan chains distributed across the RBD surface, forming a dense glycan-shield to mask epitopes beyond the ACE2 binding interface. Furthermore, to assess the impact of glycosylation modification on the recognition of other proteins, we included a mutation within the ACE2 binding interface called the mutant negative control (Mnc) (**Figures 1C, D**). The addition of a glycan chain through this mutation is expected to disrupt the recognition of the RBD by hACE2.

To enhance the immune response elicited by the RBD antigen, which has a relatively low molecular weight, we constructed an RBD-Fc fusion protein by linking the human IgG1 Fc region to the C-terminus of RBD using a GS linker (**Figure 1E**). This fusion protein results in the dimerization of RBD through a disulfide bond within the human IgG1 Fc region, increasing the size of the antigenic protein and thus improving its recognition by the immune system. Additionally, the Fc fragments facilitate antigen presentation and promote a stronger immune response, while extending the half-life of the RBD antigen *in vivo* (34).

### 3.2 Characterization of RBD mutations with single glycan modification

To evaluate the impact of each mutation or glycan modification on the expression and characteristics of the RBD, we initially constructed RBD mutants with single glycosylation mutations and expressed them in HEK293T cells. Protein tests revealed that RBD mutants with glycosylation mutations M1, M2, M3, M7, and Mnc were expressed successfully, while RBD mutants M4, M5, and M6 failed to express (data not shown). Interestingly, all three mutations that resulted in failed expression were located on a relatively flat surface comprising a portion of the RBM (residues 458-471) and the α3-β4 loop (residues 426-430) (**Figure 1C**) (32), suggesting that this region is particularly sensitive to mutations or minor structural changes, despite the absence of secondary structural elements.

Next, we expressed and purified the M1, M2, M3, and M7 mutants using affinity purification followed by size exclusion chromatography (SEC). The purified proteins exhibited high purity and homogeneity (**Supplementary Figure 1**), and their elution profile from a Superdex 200 10/300 GL column indicated dimerization of the RBD-Fc proteins, consistent with our prediction. Reduced SDS-PAGE analysis of the purified RBD-Fc proteins displayed an apparent molecular weight of approximately 60 kDa (**Supplementary Figure 1**), significantly larger than the theoretical molecular weight of approximately 50 kDa, suggesting glycosylation modifications on the single mutated RBD proteins.

To further confirm the glycan modifications of the RBD-Fc mutants, we performed liquid chromatography-mass spectrometry (LC-MS) analysis. PNGase F (36 kDa), an amidase capable of cleaving N-linked glycoproteins between the innermost glycosyl group and asparagine residues, was used for digestion. Following digestion with PNGase F, the RBD-Fc mutants exhibited two smaller bands on SDS-PAGE (**Supplementary Figure 2A**). The smallest band, with an apparent molecular weight of approximately 50 kDa, was considered the product of complete deglycosylation and was used for subsequent MS detection. The MS results confirmed the successful introduction of glycan at the sites of M1, M2, M3, M7, and Mnc (**Supplementary Figures 2B-F**). Furthermore, the glycan-modified peptide accounted for more than 90% for the peptide containing the M7 site and over 99% for the M1, M2, M3, and Mnc sites (**Supplementary Figure 2G**), indicating a high population of glycosylation in the RBD-Fc mutants.

Next, we set out to validate the interaction between the glycan-modified RBD-Fc mutants and hACE2 using SEC (**Supplementary Figure 3**). Following a 2-hour incubation of hACE2 and each RBD-Fc mutant, the elution peak shifted forward compared to RBD-Fc or hACE2 alone (**Supplementary Figures 1, 3A, B**). The shifted peak contained both RBD-Fc and hACE2 (**Supplementary Figure 3C**), indicating the formation of stable complexes between these RBD-Fc mutants and hACE2 respectively. However, no shifted peak was observed for RBD-Fc Mnc, suggesting that the glycan added at the Mnc site effectively shielded the epitope and disrupted the interaction between RBD and hACE2. To further measure the binding affinity between the RBD-Fc mutants and hACE2, we performed surface plasmon resonance (SPR) analysis (**Supplementary Figure 4**). The binding affinity between RBD-Fc M1, M2, M3, and M7 and hACE2 ranged from 57 to 75 nM, similar to that of wildtype RBD-Fc and hACE2 (94 nM). Consistent with the SEC result, no interaction was detected between RBD-Fc Mnc and hACE2.

Additionally, we assessed the interaction between the RBD-Fc mutants and cell-surface-expressed hACE2 using flow cytometry (**Supplementary Figure 5**). RBD-Fc mutants M1, M2, M3, and M7, along with wtRBD, displayed robust binding to ACE2. In contrast, the binding of RBD-Fc Mnc to ACE2 was notably diminished. Taken together, our data proved that glycan masking is effective in blocking protein-protein interaction (Mnc no longer binds to ACE2.) and the masking effect is specific (M1, M2, M3, and M7 bind to ACE2 well).

### 3.3 Construction of glycan-shield RBD with multiple glycan modifications

**Figure 2:**
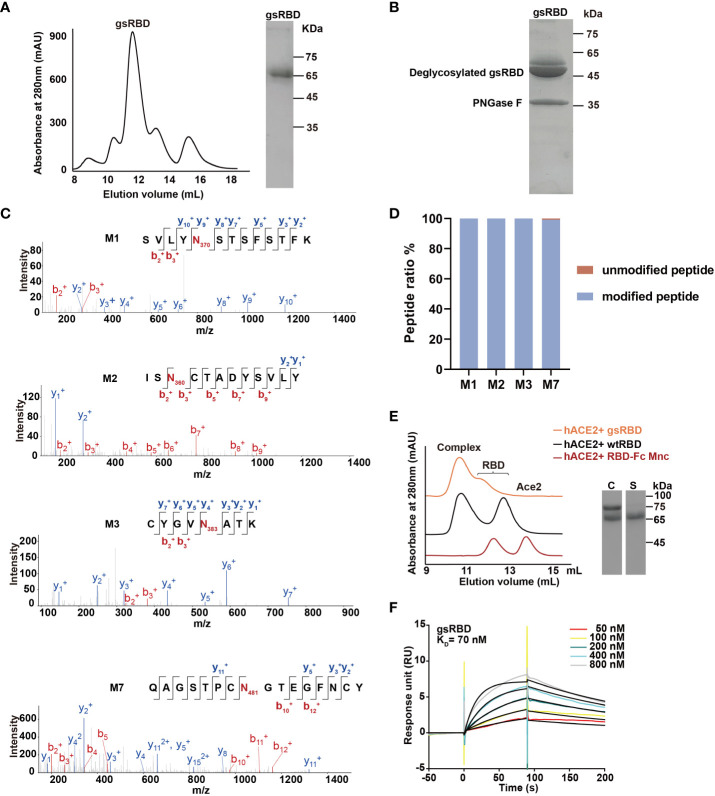
Purification and characterization of gsRBD. **(A)** Chromatogram showing the elution profile of gsRBD from a Superdex 200 10/300 GL column. SDS-PAGE analysis of the peak elution is displayed on the right. **(B)** SDS-PAGE analysis of gsRBD after digestion with PNGase F. **(C)** LC-MS analysis depicting the glycosylation status at the designed sites. The intensity profiles of fragment ions derived from peptide parent ions, including the M1, M2, M3, and M7 site of gsRBD, are presented individually. The N-glycosylation sites are highlighted in red. **(D)** Quantification of the glycan content at each site based on LC-MS analysis. Ratios of peak areas between peptides with glycan modifications and peptides without glycan modifications at the M1, M2, M3 and M7 sites are calculated and displayed. **(E)** Detection of the interaction between gsRBD and hACE2 using SEC. SDS-PAGE analysis of the eluted samples is shown on the right. C, complex. S, gsRBD. **(F)** Determination of the interaction between gsRBD and hACE2 using SPR.

Since the mutations M1, M2, M3, and M7 successfully introduced a high population of glycan chains without affecting expression and interaction with hACE2, we combined them to construct a multiple mutant RBD-Fc, named the glycan-shield RBD (gsRBD). The gsRBD was successfully expressed in HEK293T cells and purified to a high level of purity (**Figure 2A**). It eluted at approximately 12 mL from the SEC column, indicating a forward shift compared to the single mutants, and SDS-PAGE analysis displayed a larger apparent molecular weight compared to the single mutants (**Figure 2A**, **Supplementary Figure 1**), confirming the successful addition of glycan to the RBD protein at multiple positions.

The glycan modification of gsRBD was further analyzed by MS after PNGase F digestion (**Figures 2B, C**). Similar to the single RBD mutants, two bands with an apparent molecular weight of approximately 50 kDa were obtained after PNGase F digestion, indicating the complete removal of glycan chains from gsRBD. MS analysis further confirmed the high population of glycan modification at the M1, M2, M3, and M7 sites (**Figure 2D**). SEC analysis indicated that gsRBD formed a stable complex with the hACE2 protein (**Figure 2E**), and SPR analysis demonstrated that the binding affinity between gsRBD and hACE2 was 70 nM (**Figure 2F**), similar as that between wtRBD and hACE2. Furthermore, gsRBD showed a strong binding to the cell-surface-expressed hACE2 (**Supplementary Figure 5B**).

In summary, we successfully designed and produced a glycan-modified gsRBD, which was glycosylated at four additional sites beyond the native glycosylation sites. The introduction of glycan modification did not affect its recognition and binding to hACE2.

### 3.4 Immunogenicity of gsRBD in mice

**Figure 3:**
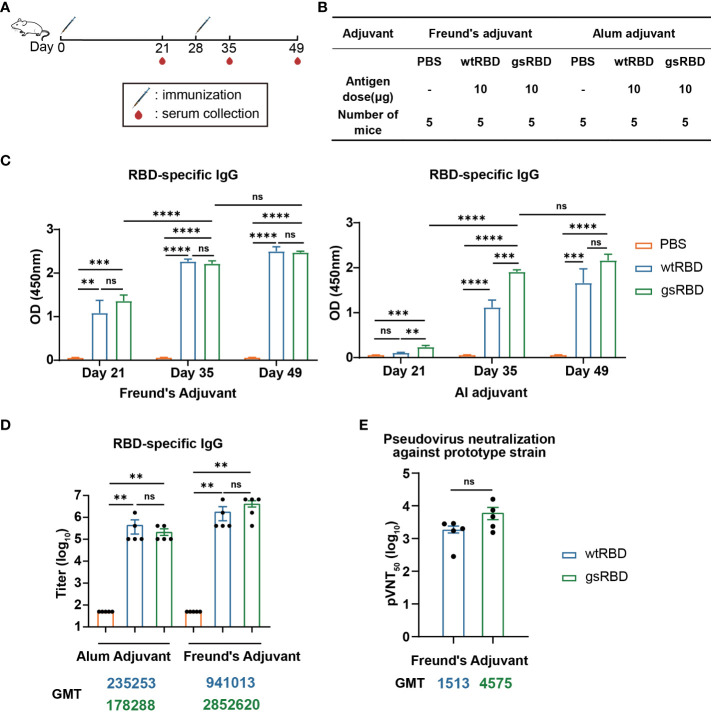
Antibody response elicited by gsRBD against the SARS-CoV-2 prototype. **(A, B)** Schematic representation and experimental design of immunization in BALB/c mice. BALB/c mice were immunized with gsRBD combined with either Freund’s adjuvant or Alum adjuvant. wtRBD and PBS served as positive and negative controls, respectively. Each group consisted of five mice. A booster injection was administered 28 days after the primary immunization. Blood samples were collected on day 21, 35, and 49 after the primary immunization. **(C)** IgG response against RBD of the SARS-CoV-2 prototype in sera collected at different time points. ELISA was performed to measure the binding of mouse sera (n=5) collected on day 21, 35, and 49, diluted at 1: 10,000, to RBD. Data are presented as mean ± SEM. ns, P > 0.05. *, P < 0.05. **P < 0.01. ***P < 0.001. ****P < 0.0001. P-values were calculated using one-way ANOVA followed by Tukey’s multiple comparison test. **(D)** IgG titers against RBD of the SARS-CoV-2 prototype. Analysis was performed on mouse sera (n=5) collected on day 49. The GMT (geometric mean titer) values for each group are shown below the graph. The color of the GMT values corresponds to the color of the bars, with yellow, blue, and green representing the PBS, wtRBD, and gsRBD groups, respectively. Data are presented as mean ± SEM. ns, P > 0.05. *P < 0.05. **P < 0.01. ***P < 0.001. P-values were calculated using two-tailed Mann–Whitney tests. **(E)** Neutralizing IgG titers against the SARS-CoV-2 prototype pseudovirus. The y-axis represents the serum dilution at which 50% inhibition was observed. The GMT values for each group are shown below the graph. The color of the GMT values corresponds to the color of the bars, with blue and green representing the wtRBD and gsRBD groups, respectively. Data are presented as mean ± SEM. ns, P > 0.05. *P < 0.05. **P < 0.01. ***P < 0.001. P-values were calculated using two-tailed Mann–Whitney tests.

To evaluate the immunogenicity of gsRBD, we conducted immunization experiments in BALB/c mice using gsRBD as the antigen protein, with wtRBD as the positive control (35) and PBS as the negative control. Two adjuvants were employed to boost the immune response to RBD: Freund’s adjuvant and alum adjuvant. Freund’s adjuvant is extensively utilized in animal experiments and is able to elicit a robust immune response (36), while alum adjuvant is the most widely used adjuvant in human applications (37). Each antigen protein was administered at a dosage of 10 μg, mixed with either Freund’s adjuvant or alum adjuvant for immunization. Each group consisted of five mice, and the mice were immunized twice at a 4-week interval (**Figures 3A, B**). In the Freund’s adjuvant group, mice were initially immunized with Complete Freund’s Adjuvant and subsequently boosted with Incomplete Freund’s Adjuvant. Mouse sera were collected three weeks after the primary immunization (Day 21), as well as one week (Day 35) and three weeks (Day 49) after the boost immunization.

To analyze the immune response at different stages of immunization, we performed enzyme-linked immunosorbent assay (ELISA) to detect the binding between RBD and 10,000-fold diluted sera collected on Day 21, 35, and 49, respectively (**Figure 3C**). In the Freund’s adjuvant group, the RBD-specific IgG levels in mice injected with both gsRBD and wtRBD significantly increased after the primary immunization. They further increased one week after the boost immunization, and no further increase was observed with the sera collected three weeks after the boost immunization. Importantly, no significant difference was observed between the gsRBD and wtRBD groups. However, in the alum adjuvant group, gsRBD elicited significantly higher RBD-specific IgG levels than wtRBD three weeks after the primary immunization and one week after the boost immunization. These data suggest that when combined with the Freund’s adjuvant and the alum adjuvant, respectively, gsRBD elicited a comparable or faster immune response compared to wtRBD.

Furthermore, we measured the titer of RBD-specific IgG in the sera collected on Day 49 (**Figure 3D**, **Supplementary Figure 6A**). In the alum adjuvant group, the geometric mean titer (GMT) of the gsRBD group was comparable to that of the wtRBD group. In the Freund’s adjuvant group, the titer of RBD-specific IgG antibodies elicited by gsRBD was approximately 3-fold higher (although not statistically significant) than that of the wtRBD group. These results indicate that both wtRBD and gsRBD can induce high levels of IgG titers in mice, and the glycan shield of RBD does not compromise the immunogenicity of wtRBD.

Moreover, we evaluated the neutralizing capacity using a pseudo-typed lentivector enveloped with the spike protein of the SARS-CoV-2 prototype strain. The GMT of 50% pseudo-virus neutralization titer (pVNT_50_) in the sera of mice immunized with gsRBD was 3-fold higher than that in the sera of mice immunized with wtRBD, although the difference was not statistically significant (**Figure 3E**, **Supplementary Figure 6B**). This result suggests that the glycan shield added to gsRBD effectively masks non-neutralizing immune-dominant epitopes on the surface of RBD. Consequently, the immune response to gsRBD is focused on the unmasked neutralizing epitopes, leading to a higher titer of neutralizing antibodies.

### 3.5 gsRBD induced the production of broad neutralizing antibodies against Beta, Delta, and Omicron variants

Since the outbreak, multiple variants of SARS-CoV-2 have emerged (38–40), and mutations in various amino acids have increased the adaptability and immune evasion capabilities of the virus (41, 42). The Omicron (B.1.1.529) variant, for instance, carries 37 mutations in the S protein, predominantly located in neutralizing epitopes, which diminishes the protective effect of the majority of currently available vaccines (43, 44). Therefore, it is crucial to assess the neutralizing antibodies induced by gsRBD against SARS-CoV-2 variants.

**Figure 4:**
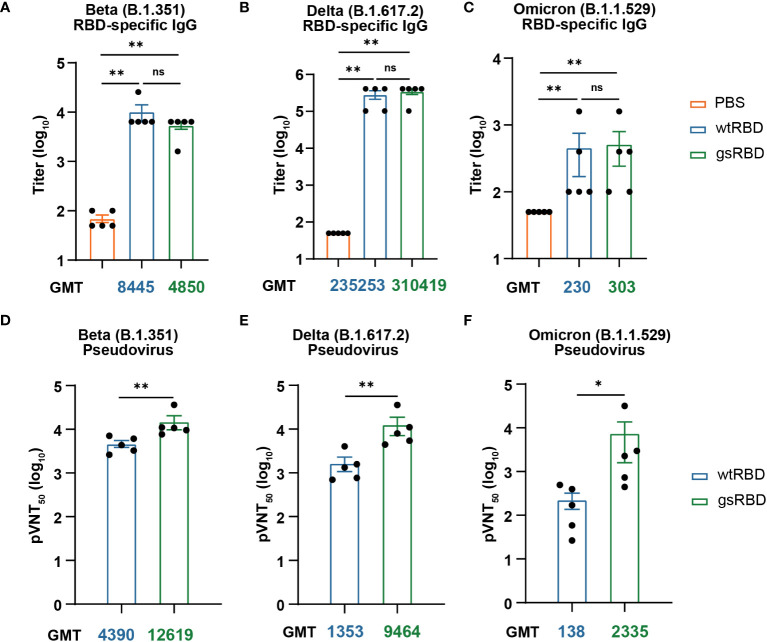
Antibody response induced by gsRBD against the SARS-CoV-2 variants. **(A-C)** IgG titers against RBD of the SARS-CoV-2 Beta **(A)**, Delta **(B)**, and Omicron **(C)** variants. Analysis was performed on sera collected from mice (n=5) immunized with Freund’s adjuvant on day 49. The GMT (geometric mean titer) values for each group are indicated below the graph. The color of the GMT values corresponds to the color of the bars, with blue and green representing the wtRBD and gsRBD groups, respectively. Data are presented as mean ± SEM. ns, P > 0.05. *P < 0.05. **P < 0.01. P-values were calculated using two-tailed Mann–Whitney tests. **(D-F)** Neutralizing IgG titers against the pseudoviruses of SARS-CoV-2 Beta **(D)**, Delta **(E)**, and Omicron **(F)** strains. Analysis was performed on sera collected from mice (n=5) immunized with Freund’s adjuvant on day 49. The y-axis represents the serum dilution at which 50% neutralization was observed. The GMT values for each group are shown below the graph. The color of the GMT values corresponds to the color of the bars, with blue and green representing the wtRBD and gsRBD groups, respectively. Data are presented as mean ± SEM. ns, P > 0.05. *P < 0.05. **P < 0.01. P-values were calculated using two-tailed Mann–Whitney tests.

Firstly, we measured the titers of IgG antibodies against the RBDs of the Beta (B.1.351), Delta (B.1.617.2), and Omicron (B.1.1.529) variants using the Day 49 sera from mice immunized with gsRBD and wtRBD in combination with the Freund’s adjuvant (**Figures 4A-C**, **Supplementary Figures 7A-C**). The IgG titers of the sera against the SARS-CoV-2 variants were lower compared to the SARS-CoV-2 prototype, particularly for the Beta and Omicron variants. While a reduction in IgG titers against the Beta variant was observed in the gsRBD-immunized group compared to the wtRBD-immunized group, an increase of IgG titers (as indicated by the GMT value) against the Delta and Omicron variants was detected. However, no significant difference was observed between the gsRBD- and wtRBD-immunized groups.

We also assessed the neutralization abilities against corresponding pseudoviruses using the Day 49 sera. Remarkably, gsRBD elicited significantly higher titers of neutralizing antibodies against all three tested SARS-CoV-2 variants compared to wtRBD (**Figures 4D-F**, **Supplementary Figurse 7D-F**). The increase in neutralizing antibodies against the Beta, Delta, and Omicron pseudoviruses was approximately 3-fold, 7-fold, and 17-fold, respectively. Interestingly, both gsRBD and wtRBD induced neutralizing antibody titers against the Beta strain that were approximately 3-fold higher compared to that against the prototype (**Figures 3E**, **4D**). Notably, while the neutralizing antibody titer against the Delta variant induced by wtRBD was comparable to that against the prototype, gsRBD showed a 2-fold increase in neutralizing antibody titer against the Delta variant compared to that against the prototype (**Figures 3E**, **4E**). Importantly, the sera in the wtRBD group exhibited a significant 10-fold decrease in neutralizing ability against the Omicron variant compared to the prototype (**Figures 3E**, **4F**), indicating a substantial loss of protection against the Omicron variant. This aligns with the fact that Omicron possesses a highly potent immune evasion ability, leading to reduced effectiveness of many vaccines (45). However, the neutralizing antibody titer induced by gsRBD was only 2-fold less potent against the Omicron variant compared to the prototype (**Figures 3E**, **4F**). Moreover, the neutralizing antibody titer against the Omicron variant elicited by gsRBD was even slightly higher than that against the prototype induced by wtRBD, suggesting that potent protection against the variants could be achieved through gsRBD immunization.

We also tested the neutralizing ability of sera from gsRBD- and wtRBD-immunized mice against pseudoviruses from two other Omicron subvariants, BQ.1.1 and XBB.1. Results showed that the maximum neutralizing ability of gsRBD-immunized sera against the Omicron (BQ.1.1) pseudovirus decreased by 40%-60% compared to the Omicron (B.1.1.529) strain (**Supplementary Figures 7F**, **8**). Only two sera from the wtRBD-immunized group exhibited very weak neutralization (**Supplementary Figure 8**). In the case of the Omicron (XBB.1) pseudovirus neutralization experiment, nearly all of the sera lost their neutralizing ability (data not shown). The significant decrease in neutralization capacity against the newly-evolved subvariants is in line with the reported findings regarding neutralization by sera from vaccinated individuals and those who were infected (46). Despite the overall decline in neutralizing ability against the newly-evolved subvariants in both gsRBD- and wtRBD-immunized mice sera, gsRBD-immunized mice showed notably higher neutralizing ability compared to the wtRBD group against the Omicron (BQ.1.1) strain (**Supplementary Figure 8**).

In summary, introducing a glycan shield to RBD induced higher levels of neutralizing antibodies in mice compared to the wild-type RBD, with more significant neutralizing effects observed against the SARS-CoV-2 variants. The enhanced neutralizing ability suggested broad protection against the SARS-CoV-2 variants.

## 4 Discussion

After its emergence, SARS-CoV-2 rapidly spread worldwide, causing a devastating global pandemic with numerous severe cases and fatalities, and significantly impacting the global healthcare system. Vaccines have emerged as the most effective method for preventing SARS-CoV-2 infection, reducing the severity of the disease, and lowering mortality rates. Although currently available vaccines can effectively stimulate the production of neutralizing antibodies, their efficacy against the rapidly evolving variants, particularly the Omicron variant, is greatly diminished (45, 47). Therefore, there is a need for continuous vaccine updates, such as redesigning vaccines using variant S protein or RBD as antigens or incorporating chimeric RBD from different mutant strains, to ensure protection against broader variants (48, 49).

In this study, we described a method to enhance the production of RBD-specific neutralizing antibodies targeting a broad range of SARS-CoV-2 variants by glycan-shielding multiple non-neutralizing epitopes. To extend the glycan shield beyond the ACE2 binding interface, we designed a series of seven glycosylation mutations and assessed their expression and glycosylation efficiency (**Figure 1**). Among these mutations, four were successfully expressed, confirmed to be highly glycosylated, and demonstrated high affinity binding to ACE2 (**Supplementary Figures 1-4**). These mutations were then combined to construct the gsRBD (**Figure 2**). Immunization of mice with gsRBD induced RBD-specific IgG titers comparable to those induced by wtRBD against both the SARS-CoV-2 prototype and its variants (**Figures 3**, **4**). However, the neutralizing antibodies elicited by gsRBD exhibited significantly higher potency against the Beta, Delta, and Omicron variant pseudoviruses compared to that induced by the wtRBD (**Figure 4**), indicating that gsRBD immunization provides broad protection against the SARS-CoV-2 variants.

The strategy of introducing glycan chains to mask undesired epitopes of antigen proteins has been used for shifting, focusing and broadening the immune response (23). Recently, three groups reported the introduction of a single glycan mask to the S protein and RBD of SARS-CoV-2 to enhance neutralizing antibodies, although a comprehensive verification of the glycan modifications and characterization of the antigen proteins were absent in those studies (50–52). In one study, the introduction of seven single N-glycan sites to the full-length S gene showed that only glycan modifications at N158 (via the R158N/Y160T mutation) in the NTD and N428 (via the D428N mutation) in the RBD elicited higher neutralizing antibodies against the Alpha (B.1.1.7) and Beta (B.1.351) variants (50). However, the neutralizing antibodies induced by S-N428 against the Delta (B.1.617.2) variant were lower compared to those induced by the wild-type S antigen, indicating a selective and mild enhancement of the neutralizing ability against SARS-CoV-2 variants through glycan introduction at N428. Nevertheless, this enhancement may not be solely attributed to the “masking” effect of the introduced glycan, as the residue at position 428 is located on the buried surface of the RBD in the S trimer when the RBD is in the “down” conformation (53). Therefore, the introduction of a glycan at this position may result in steric conflicts within the S trimer, leading to a constant “up” conformation of the RBD and exposure of the ACE2-binding neutralizing epitopes. Interestingly, in our study, we also designed a glycan modification at N428 (via the D428N/F429A mutation), which, unfortunately, failed to express, possibly due to the deleterious effect of the additional F429A mutation.

In another study, an RBD subunit vaccine with a single glycan mask at N519 (via H519N/P521T or H519N/P521S mutation) demonstrated a significant enhancement of neutralizing antibodies against a broad panel of SARS-CoV-2 variants (52). Glycan masking at a closely neighboring residue, N521 (via the P521N mutation), also led to increased neutralizing antibodies against the Delta (B.1.617.2) and Gamma (P.1) variants when mice were immunized with DNA/Modified Vaccinia Ankara (MVA)-delivered RBD (51). However, when the glycan mask at N519 (via the H519N/P521T mutation) was introduced to the full-length S gene, a decrease in neutralizing ability against the SARS-CoV-2 prototype, and Alpha (B.1.1.7) and Delta (B.1.617.2) variants was observed (50). This decrease is likely due to the destabilization of the S trimer caused by the mutation or glycan modification at this site, which has little effect on the structural stability of the RBD itself. These studies suggest that the selection of the antigen is also crucial for the effectiveness of glycan masking.

The enhancement of neutralizing ability achieved by glycan masking of the N519 residue of RBD (52) is similar to the findings in our study. Both studies employed RBD-Fc as the antigen in subunit vaccines, although the immunization scheme differed. The sera collected from hACE2-Tg mice after three immunizations (52) showed comparable pVNT_50_ values against the SARS-CoV-2 prototype to those obtained after two immunizations in our study. However, the neutralizing antibodies elicited by gsRBD against the Delta and Omicron variants in our study were approximately 4.5-fold and 2.5-fold higher, respectively, compared to those induced by RBD with a glycan at N519. These findings suggest that shielding the RBD protein with multiple glycan modifications is more effective in enhancing its neutralizing ability against SARS-CoV-2 variants than a single glycan mask.

When designing a glycan shield, the selection of glycosylation sites is a critical consideration. In this study, we visually inspected the structure of RBD and selected seven sites. However, three mutations (M4, M5 and M6) located on a relatively flat surface failed to express, indicating that this region (residues 458-471 and 426-430) may be sensitive to mutations. It is worth noting that despite the rapid evolution of the SARS-CoV-2 virus, this region is highly conserved, suggesting that the design of glycan modifications should avoid targeting these highly conserved regions. Interestingly, antibodies targeting this epitope demonstrate moderate neutralizing potency with cross-reactivity within sarbecoviruses (54). Additionally, in a separate study, the introduction of glycans at non-conserved sites within the core antigenic sequence of sarbecovirus RBD effectively enhanced the presentation of conserved epitopes to the immune system. This approach demonstrated broad protective effect against pan-sarbecovirus, including SARS-CoV-1, SARS-CoV-2, WIV16, and RaTG13, in mice, rabbits, and guinea pigs (49).

Overall, our study achieved successful glycan shielding of the RBD of the S protein through introducing multiple glycan modifications, resulting in a significant enhancement of neutralizing antibodies against a broad range of SARS-CoV-2 variants. These findings not only provide an optimized antigen candidate for the design of next-generation vaccines against the SARS-CoV-2 virus but also offer guidance for the design of glycan shielding in candidate antigens. Further optimization could involve incorporating additional beneficial glycan mutations, including N519, to further enhance its protective efficacy.

## Data availability statement

The raw data supporting the conclusions of this article will be made available by the authors, without undue reservation.

## Ethics statement

The animal study was approved by Experimental Animal Ethics Committee, Institute of Radiation Medicine, Chinese Academy of Medical Sciences and Peking Union Medical College. The study was conducted in accordance with the local legislation and institutional requirements.

## Author contributions

QZ: Investigation, Writing – original draft, Methodology. YY: Investigation, Methodology, Writing – review & editing. JL: Investigation, Writing – review & editing. ZW: Methodology, Writing – review & editing. YG: Investigation, Writing – review & editing. XL: Methodology, Writing – review & editing. WM: Methodology, Writing – review & editing. JX: Methodology, Writing – review & editing. LM: Conceptualization, Writing – review & editing, Methodology. XZ: Investigation, Writing – review & editing. XW: Project administration, Resources, Writing – review & editing. XM: Conceptualization, Methodology, Project administration, Writing – review & editing. KM: Conceptualization, Investigation, Project administration, Resources, Supervision, Writing – original draft, Writing – review & editing.
