# Supplementary material for: Inducing enhanced neutralizing antibodies against broad SARS-CoV-2 variants through glycan-shielding multiple non-neutralizing epitopes of RBD
Source: Front Immunol. 2023 Dec 11;14:1259386. doi: 10.3389/fimmu.2023.1259386 (PMC10750354; doi:10.3389/fimmu.2023.1259386)
Supplement: Supplementary file 1 [file DataSheet_1.docx]

Supplementary Material

Inducing enhanced neutralizing antibodies against broad SARS-CoV-2 variants through glycan-shielding multiple non-neutralizing epitopes of RBD

## Supplementary Figures


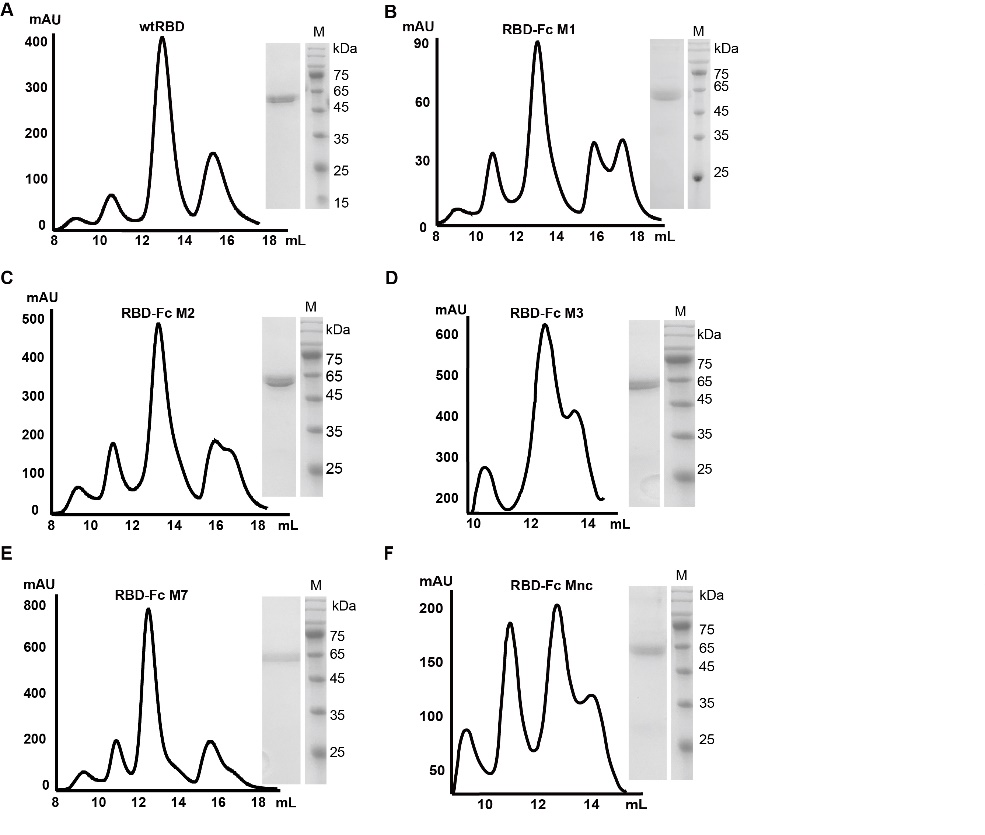


**Supplementary figure 1.** **Purification results of wtRBD (A) and RBD-Fc mutants (B-F).** The left inset in each subgraph shows the elution profile from a Superdex 200 10/300 GL column. The right inset in each subgraph is a 12% SDS-PAGE gel displaying the purified wtRBD and RBD-Fc mutants.


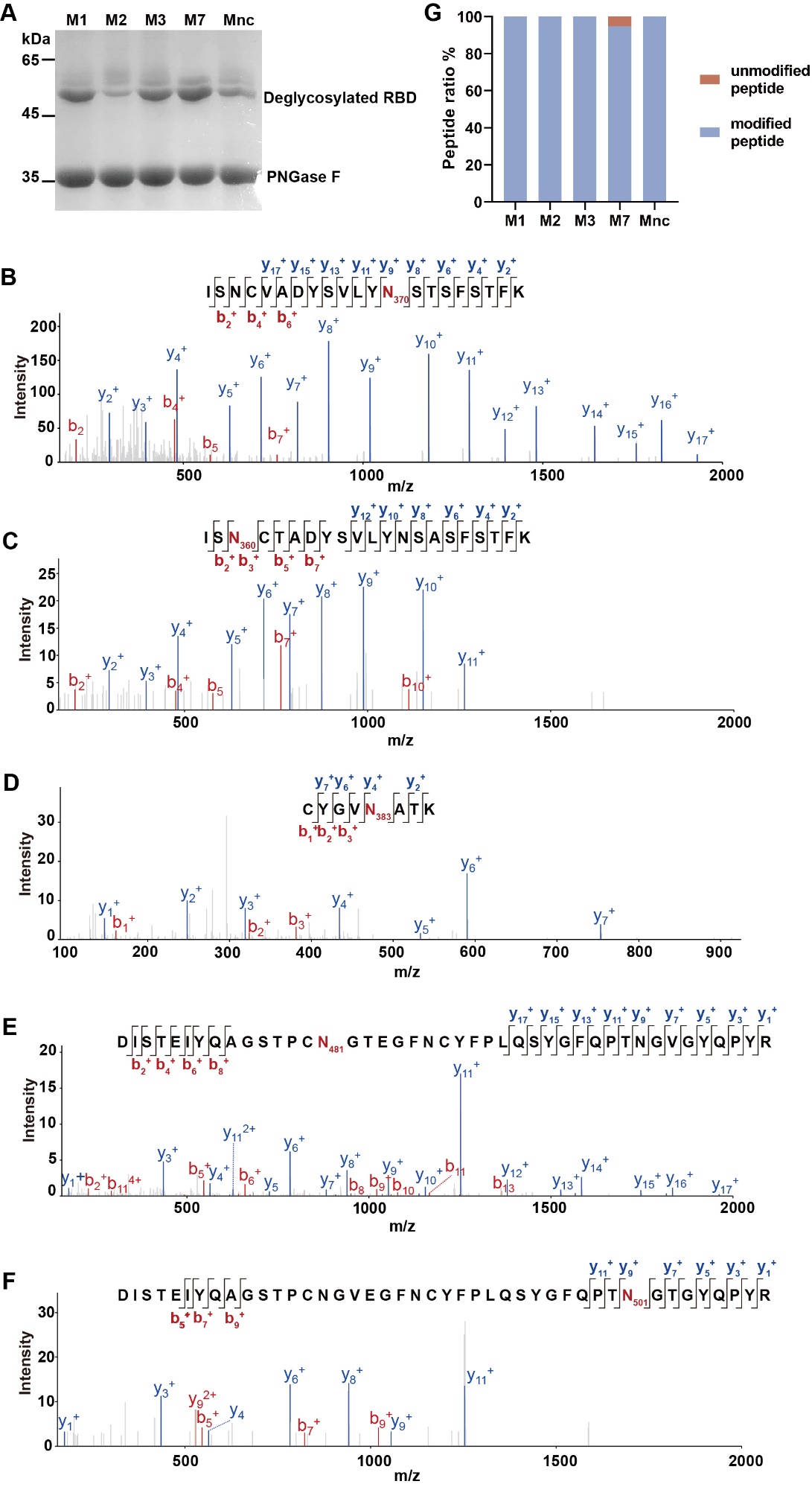


**Supplementary figure 2.** **Glycosylation detection of RBD-Fc mutations by LC-MS.** (A) SDS-PAGE analysis of RBD-Fc mutants after digestion with PNGase F. The bands corresponding to completely digested RBD-Fc mutants and PNGase F are marked. (B-F) LC-MS analysis illustrating the glycosylation status of the designed mutations. The intensity profiles of fragment ions derived from peptide parent ions, including the M1 (B), M2 (C), M3 (D), M7 (E) and Mnc (F) mutations, are presented individually. The N-glycosylation sites are highlighted in red. (G) Quantification of the glycan content for each mutation based on LC-MS analysis. Ratios of peak areas between peptides with glycan modifications and peptides without glycan modifications at the M1, M2, M3, M7, and Mnc sites are calculated and displayed.


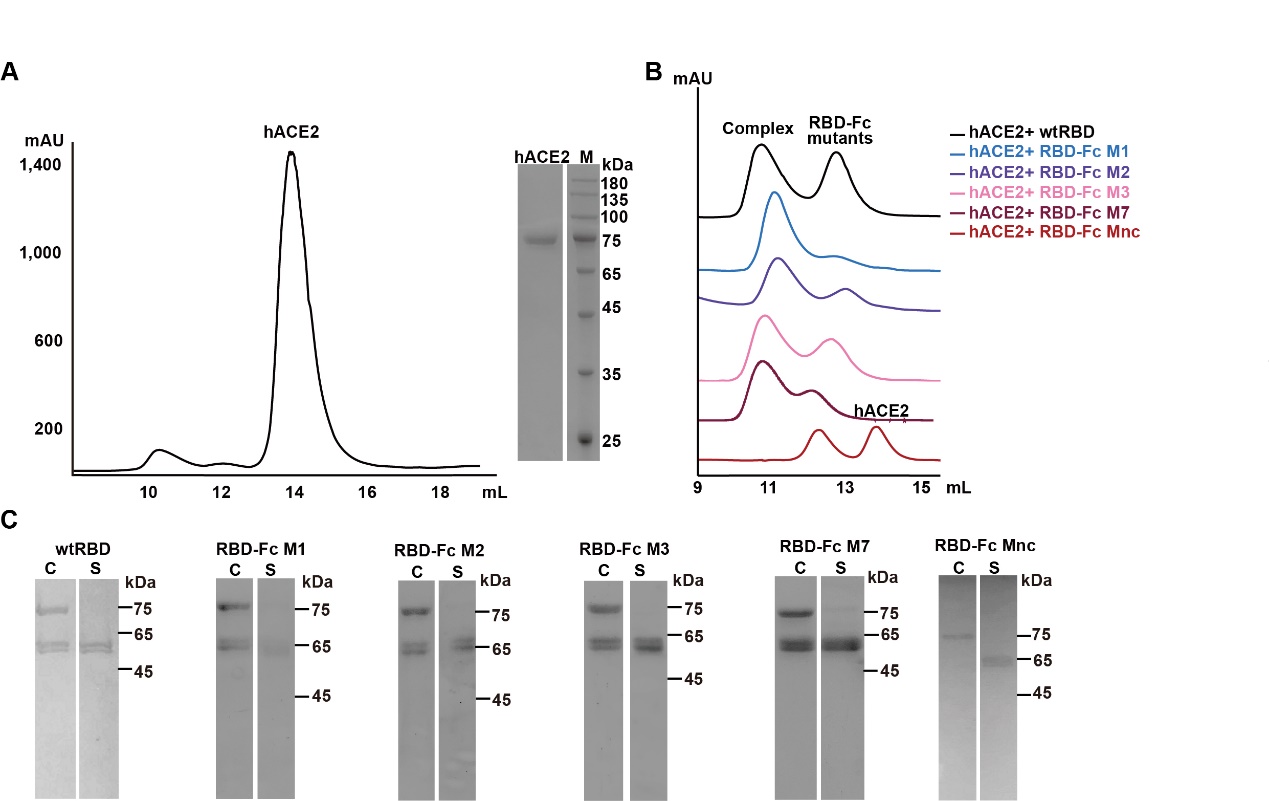


**Supplementary figure 3. Interaction detection between hACE2 and RBD-Fc mutants through SEC****.** (A) SEC purification of hACE2. The left inset is an elution profile of hACE2 purified with a Superdex 200 10/300 GL column. The right inset is a 12% SDS-PAGE gel displaying the purified hACE2. (B) SEC elution profiles of hACE2 mixed with RBD-Fc mutants. Protein mixtures were eluted from a Superdex 200 10/300 GL column. The peaks of complex, RBD-Fc, and hACE2 are markered. (C) SDS-PAGE analysis of the protein samples eluted from SEC. C, complex. S, RBD-Fc proteins.


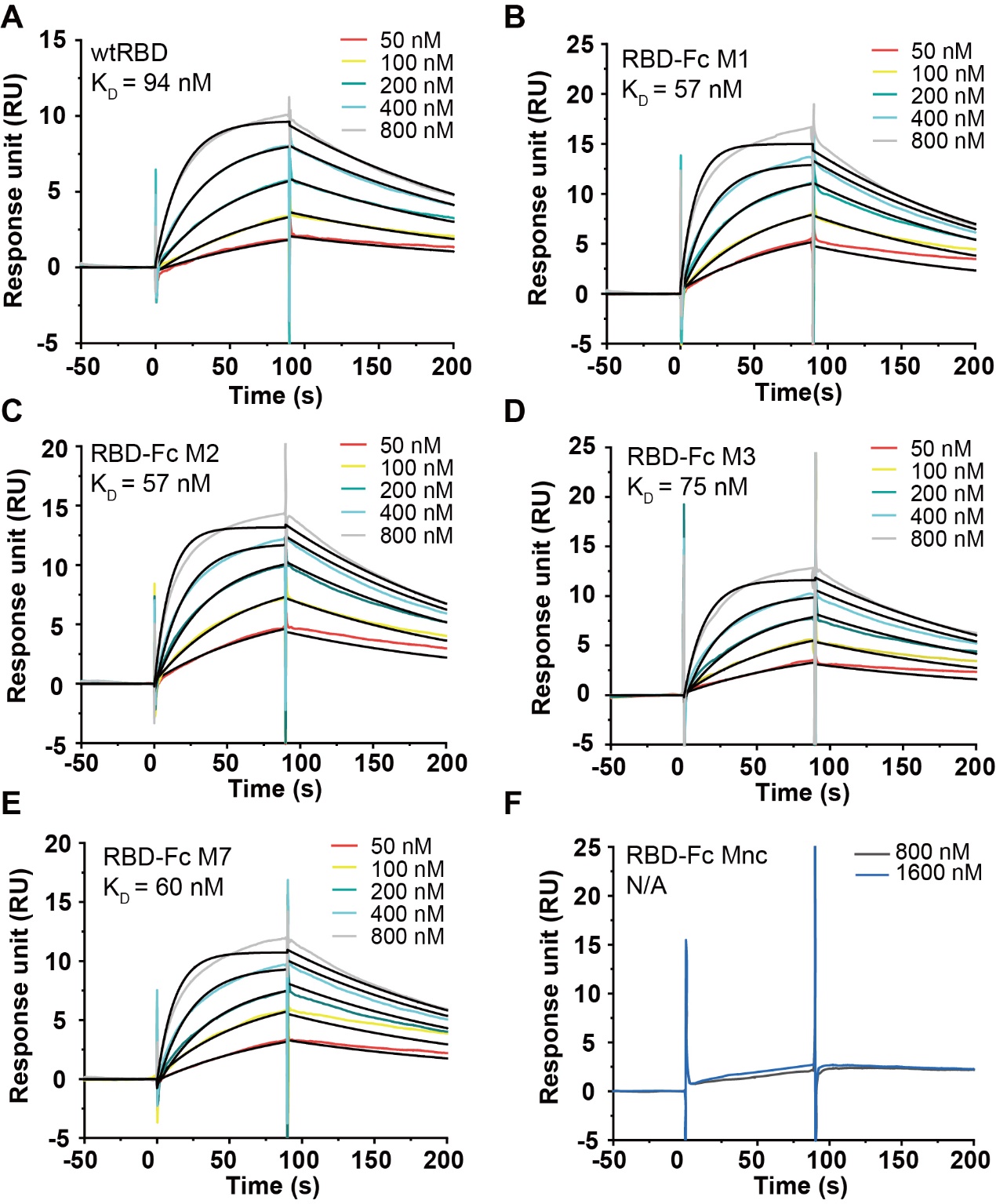


**Supplementary figure 4.** **Determination of the interaction between hACE2 and RBD-Fc mutants using SPR.** (A-F) Interactions between hACE2 and RBD-Fc WT (A), M1 (B), M2 (C), M3 (D), M7 (E), and Mnc (F) were detected. hACE2 was immobilized on a CM5 chip. The RBD-Fc mutants were serially diluted from 800 nM to 50 nM and flowed over the CM5 chip. For RBD-Fc Mnc, the concentrations of 800 nM and 1600 nM were used for interaction detection, however, no interaction was observed.

**
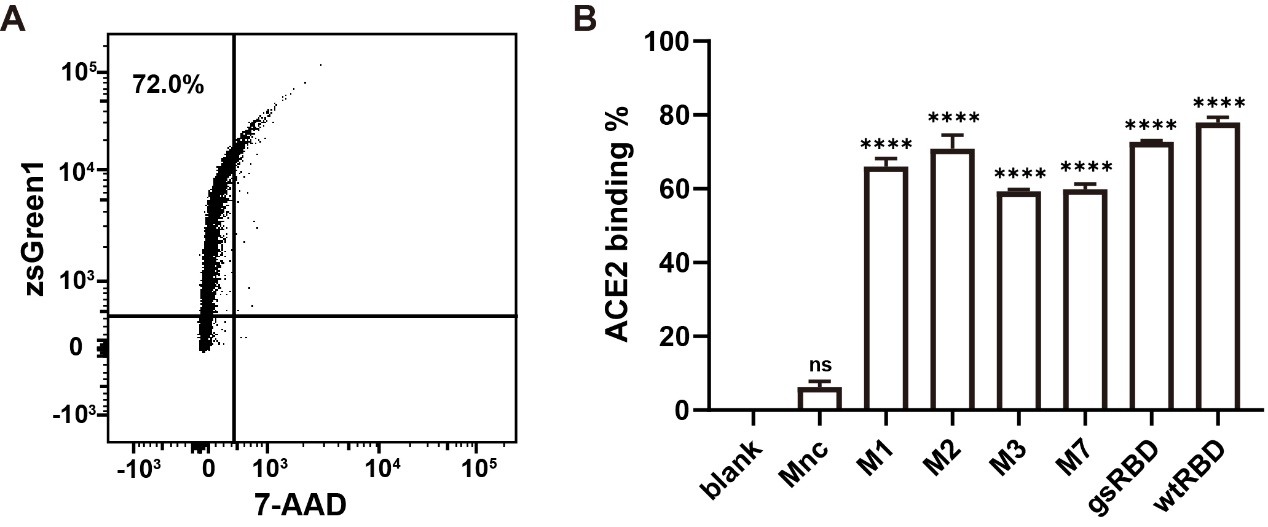
**

**Supplementary figure 5.** **Expression of hACE2 and determination of the interaction between hACE2 and RBD-Fc mutants using flow cytometry.** (A) The expression of hACE2 in HeLa cells (zsGreen1+, 7-AAD-) was analyzed using flow cytometry. (B) ACE2-expressing HeLa cells were subjected to sequential incubation with RBD proteins (1μg/ml) and goat anti-human IgG (H+L) labeled with Alexa Fluor 647. Subsequently, flow cytometry analysis was conducted. The ACE2 binding percentage was calculated as the proportion of RBD-positive cells among the ACE2-expressing cells (zsGreen1+), n=3. The data is presented as the mean ± SEM.


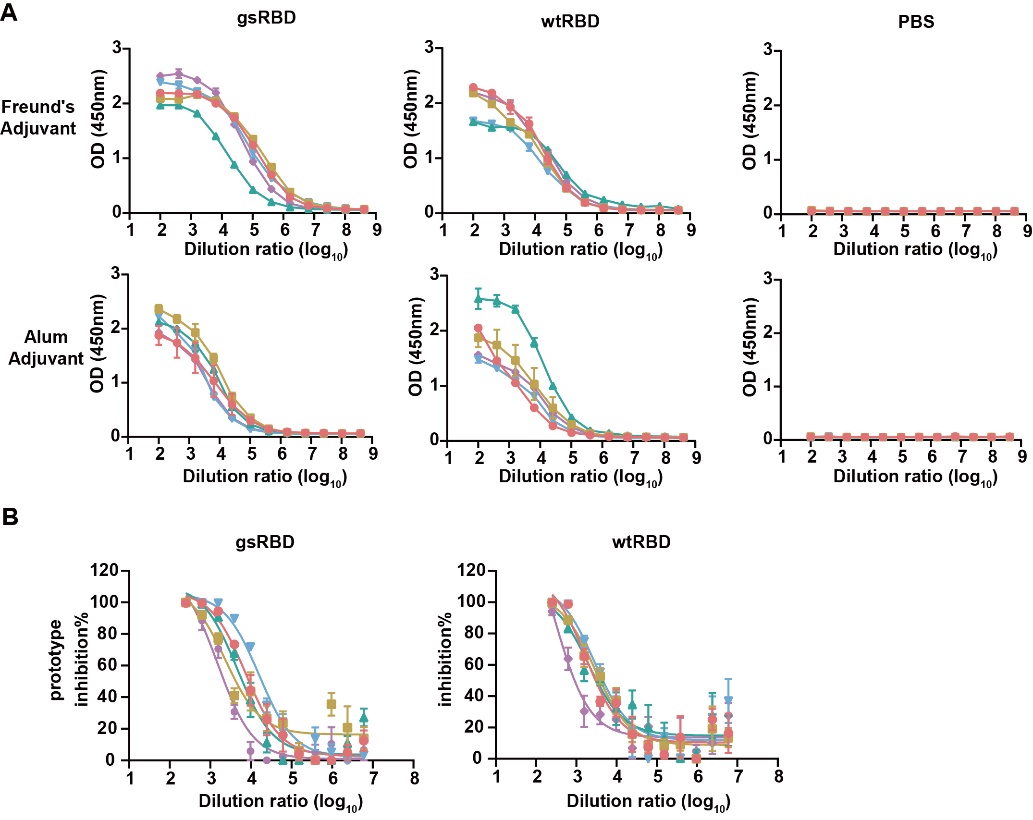


**Supplementary figure 6.** **Titration of IgG antibodies induced by gsRBD against the SARS-CoV-2 prototype, related to Figure 3.** IgG antibodies against the RBD (A) and pseudovirus (B) of the SARS-CoV-2 prototype were titrated. Analysis was performed on sera collected from mice (n=5) immunized with Freund's adjuvant and alum adjuvant (A) or Freund's adjuvant alone (B) on day 49. Each curve represents the data from an individual mouse. Measurements were performed in triplicate. Data are shown as mean ± SEM.


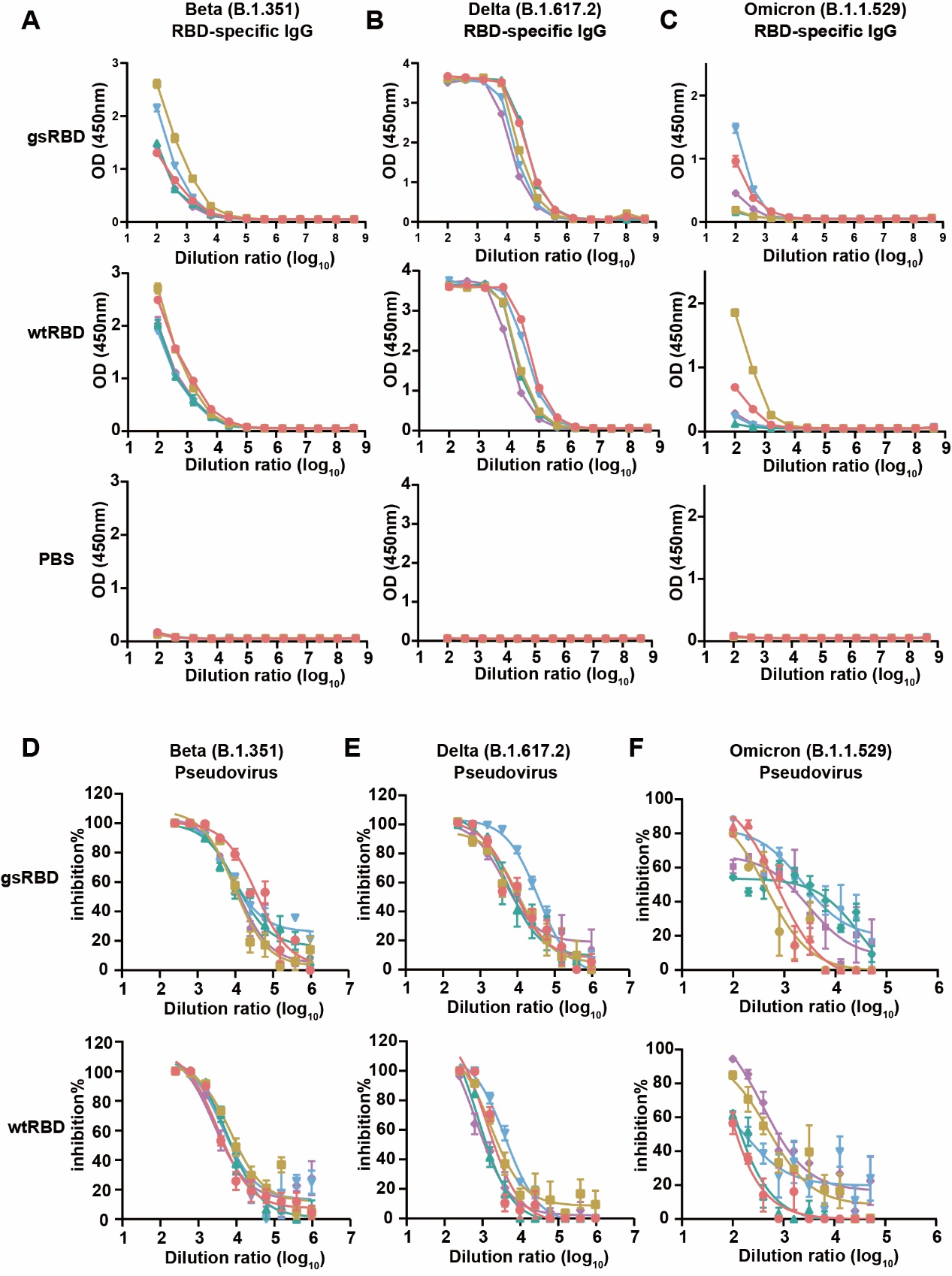


**Supplementary figure 7.** **Titration of IgG antibodies induced by gsRBD against the SARS-CoV-2 variants, related to Figure 4.** (A-C) IgG antibodies against the RBD of the SARS-CoV-2 Beta (A), Delta (B) and Omicron (C) variants were titrated. Analysis was performed on sera (n=5) collected from mice immunized with Freund's adjuvant on day 49. Each curve represents the data from an individual mouse. Measurements were performed in triplicate. Data are shown as mean ± SEM. (D-F) Neutralizing IgG antibodies against the pseudovirus of the SARS-CoV-2 Beta (D), Delta (E) and Omicron (F) variants were titrated. Analysis was performed on sera (n=5) collected from mice immunized with Freund's adjuvant on day 49. Each curve represents the data from an individual mouse. Measurements were performed in triplicate. Data are shown as mean ± SEM.

**
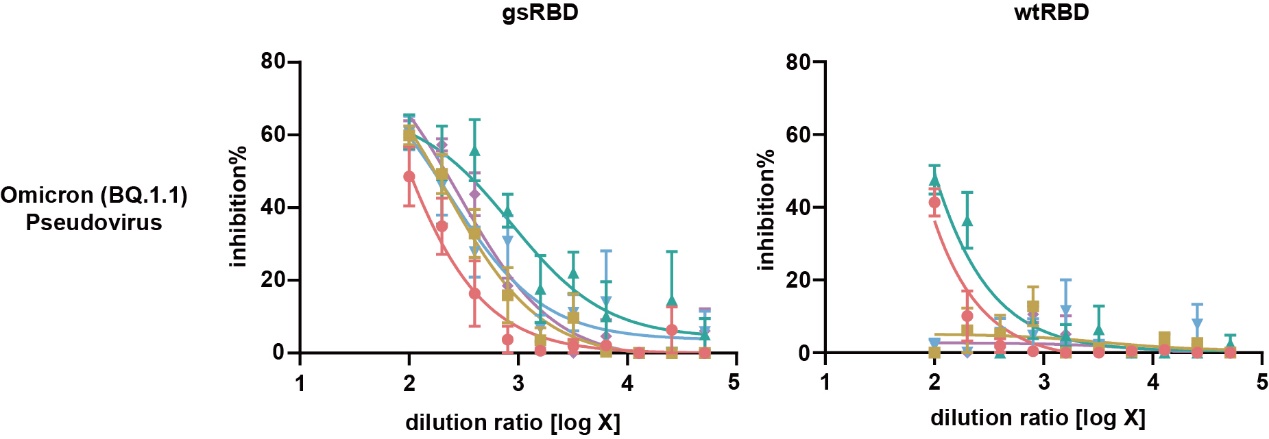
**

**Supplementary figure 8.** **Titration of IgG antibodies induced by gsRBD against the BQ.1.1 pseudovirus.** Neutralizing IgG antibodies against the pseudovirus of the Omicron subvariant BQ.1.1 were titrated. Analysis was performed on sera (n=5) collected from mice immunized with Freund's adjuvant on day 49. Each curve represents the data from an individual mouse. Measurements were performed in triplicate. Data are shown as mean ± SEM.
